# Xeno-Free Spheroids of Human Gingiva-Derived Progenitor Cells for Bone Tissue Engineering

**DOI:** 10.3389/fbioe.2020.00968

**Published:** 2020-08-19

**Authors:** Siddharth Shanbhag, Salwa Suliman, Anne Isine Bolstad, Andreas Stavropoulos, Kamal Mustafa

**Affiliations:** ^1^Department of Clinical Dentistry, Faculty of Medicine, University of Bergen, Bergen, Norway; ^2^Department of Periodontology, Faculty of Odontology, Malmö University, Malmö, Sweden; ^3^Division of Regenerative Medicine and Periodontology, University Clinics of Dental Medicine, University of Geneva, Geneva, Switzerland

**Keywords:** platelet lysate, mesenchymal stromal cells, gingival stem cells, spheroid culture, bone tissue engineering, regenerative medicine

## Abstract

Gingiva has been identified as a minimally invasive source of multipotent progenitor cells (GPCs) for use in bone tissue engineering (BTE). To facilitate clinical translation, it is important to characterize GPCs in xeno-free cultures. Recent evidence indicates several advantages of three-dimensional (3D) spheroid cultures of mesenchymal stromal cells (MSCs) over conventional 2D monolayers. The present study aimed to characterize human GPCs in xeno-free 2D cultures, and to test their osteogenic potential in 3D cultures, in comparison to bone marrow MSCs (BMSCs). Primary GPCs and BMSCs were expanded in human platelet lysate (HPL) or fetal bovine serum (FBS) and characterized based on *in vitro* proliferation, immunophenotype and multi-lineage differentiation. Next, 3D spheroids of GPCs and BMSCs were formed via self-assembly and cultured in HPL. Expression of stemness- (SOX2, OCT4, NANOG) and osteogenesis-related markers (BMP2, RUNX2, OPN, OCN) was assessed at gene and protein levels in 3D and 2D cultures. The cytokine profile of 3D and 2D GPCs and BMSCs was assessed via a multiplex immunoassay. Monolayer GPCs in both HPL and FBS demonstrated a characteristic MSC-like immunophenotype and multi-lineage differentiation; osteogenic differentiation of GPCs was enhanced in HPL vs. FBS. CD271^+^ GPCs in HPL spontaneously acquired a neuronal phenotype and strongly expressed neuronal/glial markers. 3D spheroids of GPCs and BMSCs with high cell viability were formed in HPL media. Expression of stemness- and osteogenesis-related genes was significantly upregulated in 3D vs. 2D GPCs/BMSCs; the latter was independent of osteogenic induction. Synthesis of SOX2, BMP2 and OCN was confirmed via immunostaining, and *in vitro* mineralization via Alizarin red staining. Finally, secretion of several growth factors and chemokines was enhanced in GPC/BMSC spheroids, while that of pro-inflammatory cytokines was reduced, compared to monolayers. In summary, monolayer GPCs expanded in HPL demonstrate enhanced osteogenic differentiation potential, comparable to that of BMSCs. Xeno-free spheroid culture further enhances stemness- and osteogenesis-related gene expression, and cytokine secretion in GPCs, comparable to that of BMSCs.

## Introduction

Adult mesenchymal stromal cells (MSCs) are increasingly being used in bone tissue engineering (BTE) for the reconstruction of clinically challenging bone defects. MSCs were originally identified in the bone marrow (BMSCs), and these are still the most frequently tested cells in clinical studies (Friedenstein et al., 1968; Pittenger et al., 2019). However, the yield of BMSCs obtained from the marrow mononuclear cell fraction is relatively low (≤0.01%) (Pittenger et al., 1999). Moreover, considerable donor-related variations in BMSCs, in addition to the morbidity associated with bone marrow harvesting, have prompted the investigation of ‘MSC-like’ cells from other, relatively less invasive, tissue sources (Mohamed-Ahmed et al., 2018; Wilson et al., 2019).

Oral tissues, such as dental pulp, mucosa, periodontal ligament (PDL) and gingiva, represent alternative sources of ‘MSC-like’ progenitor cells (Sharpe, 2016). Gingiva, in particular, can be harvested with minimal morbidity and rapid scarless healing, and is reported to contain a subpopulation of multipotent progenitor cells (GPCs) (Fournier et al., 2010; Mitrano et al., 2010). GPCs demonstrate the characteristic MSC-phenotype, immunomodulatory properties, and multi-lineage differentiation, possibly owing to their neural crest origins (Xu et al., 2013). Notably, GPCs have demonstrated superior properties in comparison to other MSCs *in vitro* (Yang et al., 2013; Sun et al., 2019), and the ability to regenerate bone *in vivo* (Wang et al., 2011; Ge et al., 2012). However, in all of these studies, GPCs were cultured in xenogeneic media.

A critical aspect in the clinical translation of MSC-based therapies is the use of safe and standardized culture conditions. Although commonly used for MSC expansion, several limitations of xenogeneic fetal bovine serum (FBS) supplementation have been highlighted, and current recommendations from health authorities advocate the use of ‘xeno-free’ protocols whenever possible (Bieback et al., 2019). Accordingly, xeno-free alternatives to FBS, such as human platelet lysate (HPL), have emerged (Shanbhag et al., 2017). HPL is shown to be comparable, and often superior, to FBS for the proliferation and multi-lineage differentiation of MSCs from various tissues (Burnouf et al., 2016). Moreover, MSCs expanded in HPL demonstrate enhanced osteoblastic differentiation, suggesting particular benefits for BTE (Shanbhag et al., 2017). However, no studies have yet reported on HPL-cultured GPCs.

In order to obtain clinically relevant cell numbers, current strategies demand the large-scale *ex vivo* expansion of MSCs, most commonly via plastic adherent/monolayer culture. However, this two-dimensional (2D) culture system is not representative of the 3D *in vivo* microenvironment (Sart et al., 2014; Petrenko et al., 2017). Moreover, expansion of MSCs via serial passaging in plastic-adherent cultures may alter their phenotype and diminish their regenerative and immunomodulatory potential (Follin et al., 2016; Ghazanfari et al., 2017). In contrast, the self-assembly or spontaneous aggregation of MSCs into 3D structures, mediated by unique cell-cell and cell-extracellular matrix (ECM) interactions, biomechanical cues and signaling pathways, more closely simulates their *in vivo* microenvironment or *niche* (Ahmadbeigi et al., 2012; Sart et al., 2014). The cytoskeletal changes induced by 3D culture have also been linked to ‘mesenchymal cell condensation’ (MCC) – a critical event during embryonic skeletal development via endochondral ossification, which can be recapitulated *ex vivo* (Hall and Miyake, 2000; Kale et al., 2000; Facer et al., 2005; Kim and Adachi, 2019).

While a majority of the literature is focused on BMSCs, 3D cultures have also been reported to enhance the survival, stemness, paracrine/immunomodulatory activity, and multi-lineage differentiation of oral tissue-derived MSCs (Zhang et al., 2012; Lee et al., 2017; Moritani et al., 2018; Subbarayan et al., 2018). However, few studies have characterized MSC spheroids in xeno-free cultures to facilitate clinical translation (Ylostalo et al., 2017; Dong et al., 2019). Therefore, the objectives of the present study were to establish xeno-free monolayer (2D) cultures of human GPCs in HPL, and subsequently, to test their osteogenic potential in 3D spheroid cultures in comparison to BMSCs.

## Materials and Methods

### Monolayer (2D) Cell Culture

GPCs were isolated as previously described (Fournier et al., 2010). Briefly, human gingival biopsies were collected after ethical approval (Regional Ethical Committee-North, Norway, 2016-1266) and informed consent from systemically healthy patients aged 18–31 years (*n* = 5) undergoing surgery at the Department of Clinical Dentistry, University of Bergen, Bergen, Norway. From each donor, primary connective tissue-explant cultures of GPCs were established in 5% HPL (Bergenlys^®^, Bergen, Norway) and 10% FBS (GE Healthcare, South Logan, UT, United States) supplemented growth media [Dulbecco’s Modified Eagle’s medium (DMEM, Invitrogen, Carlsbad, CA, United States) with 1% antibiotics (penicillin/streptomycin; GE Healthcare)]. BMSCs (from different patients) were isolated and cultured in HPL media as previously described (Mohamed-Ahmed et al., 2018). Details of HPL production are provided in the Supplementary Data. Cells were sub-cultured and expanded in their respective growth media in humidified 5% CO_2_ at 37°C; passage 2–4 cells from at least three different donors were used in experiments. Proliferation of GPCs in HPL and FBS over 7 days was determined via an alamar blue assay (Invitrogen); at each time point, 10% vol. dye was added to the cells, incubated for 4 h and fluorescence was measured (540 Ex/590 Em).

#### Immunophenotype of 2D GPCs

The immunophenotype of HPL- and FBS-cultured GPCs was assessed by flow cytometry based on expression of specific surface antigens according to the “minimal criteria” for defining MSCs (Dominici et al., 2006). Briefly, cells in HPL and FBS were incubated with conjugated antibodies against selected ‘negative’ (CD34, CD45, HLA-DR) and ‘positive’ (CD73, CD90, CD105) MSC markers, and additionally CD271 (all from BD Biosciences, San Jose, CA, United States), following the manufacturers’ recommendations. Quantification was performed with a BD LSR Fortessa analyzer and fluorescence activated cell sorting (FACS) of CD271^+^ GPCs with a BD FACS Aria sorter (both from BD Biosciences). Data were analyzed using flow cytometry software (Flowjo v10, Flowjo, LLC, Ashland, OR, United States).

#### Gene Expression in 2D GPCs

The expression of adipogenesis- and osteogenesis-related genes (Supplementary Table 1) in HPL- and FBS-cultured GPCs after 7 days in the appropriate induction media (see below), was assessed via quantitative real-time polymerase chain reaction (qPCR) using TaqMan^®^ real-time PCR assays (Thermo Fisher Scientific, Carlsbad, CA, United States). RNA extraction and cDNA synthesis were performed as previously described (Mohamed-Ahmed et al., 2018). The expressions of the genes of interest were normalized to that of glyceraldehyde 3-phosphate dehydrogenase (GAPDH). Data were analyzed by the ΔΔ*Ct* method and results are presented as fold changes in HPL groups relative to FBS groups.

#### Adipogenic Differentiation of 2D GPCs

The ability of GPCs to differentiate into multiple stromal lineages was tested as previously described (Mohamed-Ahmed et al., 2018). Briefly, for adipogenic differentiation, cells in HPL and FBS were cultured in StemPro^®^ adipogenic differentiation medium (Invitrogen) or standard growth medium (control). After 21 days, cells were fixed with 4% paraformaldehyde (PFA) for 10 min at RT and intracellular lipid formation was assessed via Oil red O staining (Sigma-Aldrich, St. Louis, MO, United States).

#### Osteogenic Differentiation of 2D GPCs

For osteogenic differentiation, cells in HPL and FBS were cultured in osteogenic differentiation medium prepared by adding final concentrations of 0.05 mM L-ascorbic acid 2-phosphate, 10 nM dexamethasone and 10 mM β glycerophosphate (all from Sigma-Aldrich) to the respective growth media. After 21 days, cells were fixed and extracellular calcium deposition was evaluated via Alizarin red S staining (Sigma-Aldrich). The osteogenic potential of HPL-cultured GPCs was also tested on previously validated poly(L-lactide-co-ε-caprolactone) [poly(LLA-co-CL)] copolymer scaffolds (Yassin et al., 2017) (10^6^ cells/scaffold); HPL-cultured BMSCs were used as a reference. Cell attachment and spreading on the scaffolds after 24 h was observed via scanning electron microscopy (SEM; Jeol JSM 7400F, Tokyo, Japan), as previously described (Yassin et al., 2017). After 14 days of induction, Alizarin red S staining was performed as described above. In all differentiation experiments, corresponding non-induced HPL- and/or FBS-cultured cells served as controls.

#### Neurogenic Differentiation and Immunofluorescence (IF) Staining of 2D GPCs

Since FACS isolated CD271^+^ GPCs showed a neuronal-like morphology, the expression of neuronal [βIII-tubulin (TUJ1)] and glial markers [glial fibrillary acidic protein (GFAP)] was assessed via IF staining. Briefly, cells were fixed with PFA, permeabilized with 0.1% Triton X-100 and blocked with 10% goat serum in phosphate-buffered saline (PBS; Invitrogen). Cells were incubated with primary antibodies; mouse monoclonal anti-TUJ1 (Abcam, Cambridge, United Kingdom, dilution 1:100) and chicken monoclonal anti-GFAP (Abcam, dilution 1:100) overnight at 4°C. Corresponding secondary antibodies were incubated for 1 h at RT (Thermo Fisher Scientific, dilution 1:200). After washing with PBS, the nuclei were stained with 4′,6-diamidino-2-phenylindole (DAPI) (Sigma-Aldrich, dilution 1:2000). Imaging was performed using a confocal microscope (Andor Dragonfly, Oxford Instruments, Abingdon, United Kingdom).

### 3D Spheroid Culture

Formation of GPC and BMSC spheroids was assessed via two methods: mesenspheres (Isern et al., 2013) and aggregates (Baraniak and McDevitt, 2012). Briefly, dissociated passage 1–2 monolayer GPCs and BMSCs in HPL media were seeded (1000 cells/cm^2^) in low-attachment dishes (Corning^®^, Corning, NY, United States) for 7 days to obtain mesenspheres, or in microwell-patterned 24-well plates (Sphericalplate^®^, Kugelmeiers Ltd, Erlenbach, CH) for 24 h to obtain spheroid aggregates of 1000–2000 cells. The novel design of these microwell plates was optimized for embryoid body formation (Silin, 2012). Since aggregate spheroids could be formed more predictably than mesenspheres, only the former were used in subsequent experiments. Cell viability in spheroids was assessed after 7 days via a live/dead assay (Thermo Fisher Scientific). Hereafter, the terms 2D or monolayer culture and 3D or spheroid culture are used interchangeably throughout the manuscript.

#### Gene Expression and Osteogenic Differentiation in 3D Spheroids

The expression of pluripotency/stemness-related genes (Supplementary Table 1) was assessed in 3D and 2D GPCs and BMSCs after 7 days of suspension and adherent culture, respectively, via qPCR. Similarly, the expression of osteogenesis-related genes (Supplementary Table 1) was assessed after 7 days in standard (non-induced) and osteogenically induced cultures (as described above). Gene expression experiments were performed using spheroids and monolayers generated from both independent and pooled donor-cells and data are presented as fold changes in 3D groups relative to 2D groups. Protein expression of osteogenic markers was determined after 14 days via IF (see below). Alizarin red S staining was performed after 21 days to detect mineralization in induced and non-induced spheroids and monolayers; spheroids were stained in suspension, and following paraffin embedding and histological sectioning (3–5 μm).

#### IF Staining in 3D Spheroids

The protein expression of stemness [sex determining region Y-box 2 (SOX2)] and osteogenic markers [bone morphogenetic protein 2 (BMP2), osteocalcin (OCN)] was assessed in GPC and BMSC spheroids after 10 or 14 days of suspension culture via IF staining. The primary antibodies rabbit polyclonal anti-SOX2 (Abcam, dilution 1:1000), mouse monoclonal anti-BMP2 (Bio-Techne, Abingdon, United Kingdom, dilution 1:100), and rabbit polyclonal anti-OCN (Abcam, dilution 1:100) were incubated ON at 4°C. Corresponding secondary antibodies were incubated for 1 h at RT (Thermo Fisher Scientific; dilution 1:200), and nuclei were stained with DAPI (Sigma-Aldrich; dilution 1:2000) before imaging with a confocal microscope (Andor Dragonfly). Cell autofluorescence and non-specific staining was confirmed in control samples incubated with neither or only secondary antibodies, respectively (data not shown).

#### Multiplex Cytokine Assay

Conditioned media (CM) from 2D and 3D GPCs and BMSCs were collected after 48 h culture in HPL-free medium and the concentrations of several cytokines (Supplementary Table 2) were measured using a custom multiplex assay and a Bio-Plex^®^ 200 System (both from Bio-Rad Laboratories, CA, United States), according to the manufacturer’s instructions. Although the initial number of cells seeded in 2D and 3D cultures was the same, to account for differences in the rates of cell proliferation between the conditions, cytokine concentrations (pg/mL) were normalized to the corresponding total DNA (ng/mL). DNA quantification was performed using the Quant-IT^®^ PicoGreen dsDNA Assay Kit (Thermo Fisher Scientific) according to the manufacturer’s instructions.

### Statistical Analysis

Statistical analyses were performed using GraphPad Prism v 8.0 (GraphPad Software, San Diego, CA, United States). Data are presented as means (± SD), unless specified. Analyses of gene expression data are based on delta-CT values and results are presented as relative (log/non-linear) fold changes using scatter plots. Multiplex proteomic data are presented on a logarithmic (log_10_) scale. All other linear data are presented as bar graphs. Normality testing was performed via the Shapiro–Wilk test. The student *t*-test, Mann–Whitney *U*-test or one-way analysis of variance (ANOVA followed by a *post hoc* Tukey’s test for multiple comparisons), were applied as appropriate, and *p* < 0.05 was considered statistically significant.

## Results

### Characterization of 2D GPCs

**FIGURE 1:**
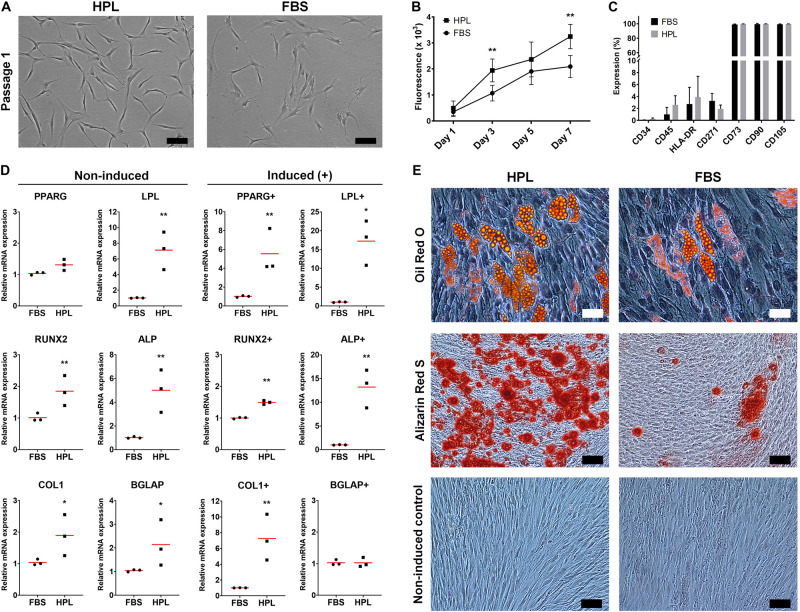
Characterization of monolayer GPCs in HPL and FBS. **(A)** Morphology of passage 1 GPCs from one representative donor; scale bars 100 μm. **(B)** Proliferation of GPCs based on metabolic activity over 7 days; data represent means ± SD (*n* = 3 donors); **p < 0.001. **(C)** Percentage expression of positive and negative surface markers based on flow cytometry; data represent means ± SD (*n* = 3 donors). **(D)** Relative expression (fold changes) of adipogenesis- and osteogenesis-related genes in GPCs after 7 days culture in growth or induction media (+). Data represent means; each symbol represents a single donor (*n* = 3 donors) based on the average of ≥ 2 experimental replicates; statistical analyses are based on delta-Ct values; *p < 0.05; **p < 0.001. **(E)** Representative images of Oil red O (adipogenic: scale bars 50 μm), Alizarin Red S (osteogenic) and control (non-induced) stained GPCs after 21 days; scale bars 100 μm.

GPCs demonstrating characteristic plastic adherence and fibroblastic morphology were isolated from gingiva explants in both HPL- and FBS-media. GPCs in HPL appeared smaller and more spindle-shaped, especially in early passages (Figure 1A), and demonstrated a higher proliferation rate (*p <* 0.05) (Figure 1B). Both HPL- and FBS-expanded GPCs demonstrated a characteristic MSC phenotype, i.e., > 95% of the cells were positive for CD73, CD90 and CD105, and < 5% of the cells expressed the hematopoietic markers CD34 and CD45; HLA-DR expression was < 8% (Figure 1C). Expression of CD271 was observed in < 5% of GPCs in both conditions.

#### Adipogenic Differentiation of 2D GPCs

GPCs in both HPL and FBS demonstrated the capacity to differentiate into adipocytes. The expression of genes associated with adipogenic differentiation, peroxisome proliferator-activated receptor-gamma (PPARG) and lipoprotein lipase (LPL), was significantly upregulated in HPL- vs. FBS-cultured GPCs after 7 days of adipogenic induction; LPL was also upregulated in non-induced HPL-cultured GPCs (*p <* 0.05; Figure 1D). Accumulation of intracellular lipid vesicles after 21 days was confirmed via Oil red O staining of GPCs in both conditions (Figure 1E). No differentiation of control cells was observed in the standard growth media.

#### Osteogenic Differentiation of 2D GPCs

GPCs in both HPL and FBS demonstrated the capacity to differentiate into osteoblasts. Genes associated with both early [runt-related transcription factor 2 (RUNX2), alkaline phosphatase (ALP)] and late osteogenic differentiation [collagen I (COL1), osteocalcin (OCN/BGLAP)] were upregulated in HPL- vs. FBS-cultured GPCs after 7 days; these genes were also upregulated in non-induced HPL-cultured GPCs (*p <* 0.05; Figure 1D). Extracellular calcium deposition was confirmed via Alizarin red S staining after 21 days; greater calcium deposition was observed in HPL-cultured GPCs (Figure 1E). Next, the osteogenic differentiation of HPL-cultured GPCs was tested on copolymer scaffolds in comparison to that of BMSCs. Cell attachment and spreading on the scaffold surface was confirmed after 24 h via SEM. After 14 days of osteogenic induction the entire scaffold surface was covered with mineralized matrix as revealed by Alizarin red S staining; staining was comparable between GPCs and BMSCs (Supplementary Figure 1).

#### Neurogenic Differentiation of 2D GPCs

**FIGURE 2:**
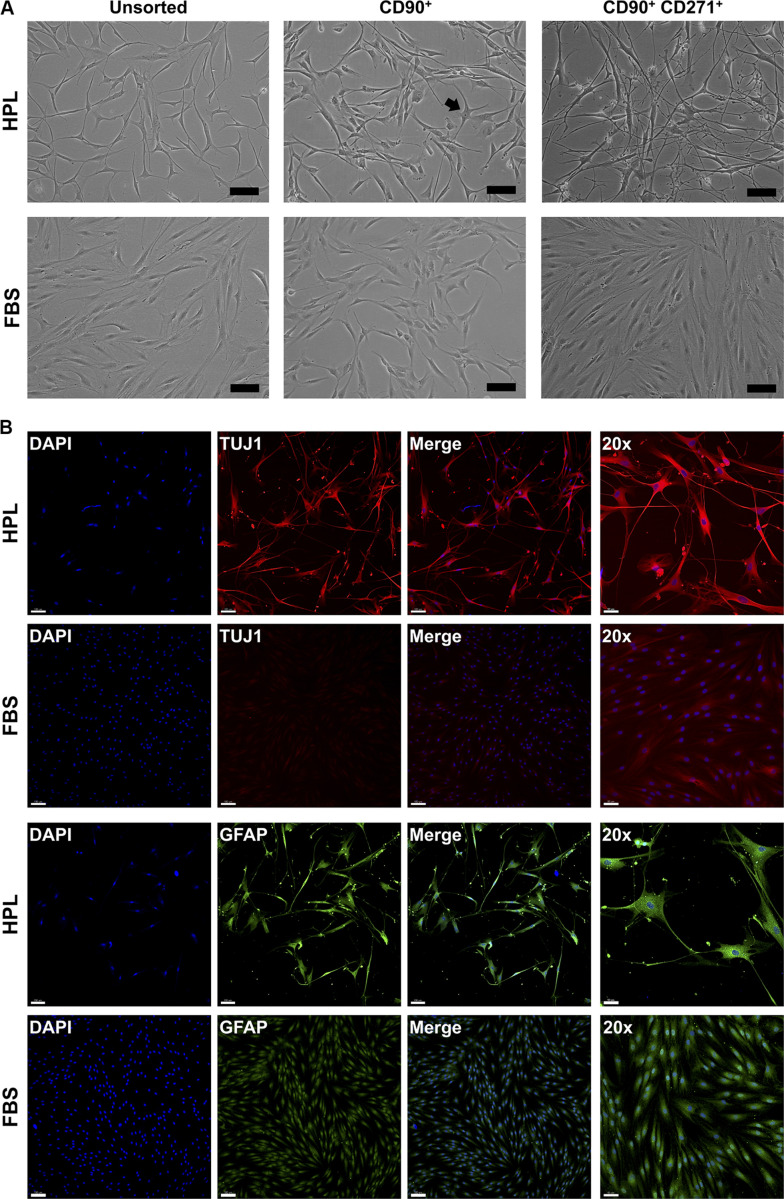
Characterization of CD271^+^ GPCs. **(A)** Selection of CD271^+^ GPCs via FACS revealed a neuronal morphology in HPL-, but not FBS-cultured cells; *unsorted* cells represent the total plastic adherent gingival cell population; CD90 was used as a ‘reference’ marker (some cells with neuronal morphology are visible – *arrow*); scale bars 100 μm. **(B)** IF staining for βIII-tubulin (TUJ1) and GFAP in CD271^+^ GPCs; scale bars 100 μm (50 μm for 20× images).

To investigate whether CD271 represents a marker to enrich osteogenic cells, CD271^+^ GPCs in HPL and FBS media were isolated via FACS. Interestingly, these cells acquired a neuronal morphology, which was more evident in HPL- than FBS-cultures (Figure 2A). Subsequently, IF staining revealed an abundant expression of neuronal (TUJ1) and glial markers (GFAP) in HPL-cultured CD271^+^ GPCs, while only a few FBS-cultured cells appeared to express these markers (Figure 2B).

### Formation and Viability of 3D Spheroids

**FIGURE 3:**
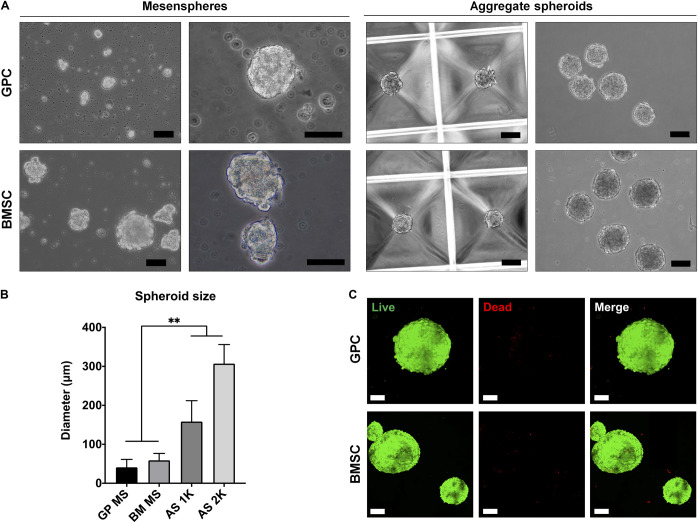
Formation of xeno-free 3D spheroids. **(A)** Representative images of GPC and BMSC mesenspheres (scale bars 50 μm) and aggregate spheroids (scale bars 100 μm). **(B)** Quantification of spheroid size in mesenspheres (MS), and aggregate spheroids of 1000 (AS 1K) and 2000 cells (AS 2K); data represent means ± SD (*n* = ≥ 10 spheres); **p < 0.001. **(C)** Viability of GPC and BMSC aggregate spheroids represented by live (green) and dead (red) cells: scale bars 100 μm.

3D spheroids of GPCs and BMSCs were formed as mesenspheres or aggregates in HPL media (Figure 3A). Since the former method relies on the self-renewal capacity of individual cells, the size and shape of mesenspheres varied considerably (ϕ < 100 μm) and the frequency of sphere formation was low; sphere formation in GPCs was considerably lower than in BMSCs. In contrast to mesenspheres, highly consistent spheroids of GPCs and BMSCs were obtained via spontaneous aggregation in microwells (∼1000 cells/spheroid, ϕ 100–300 μm; Figure 3B). Viability of a majority of cells within the aggregate spheroids was confirmed via live/dead staining (Figure 3C).

#### Gene Expression and Osteogenic Differentiation in 3D Spheroids

**FIGURE 4:**
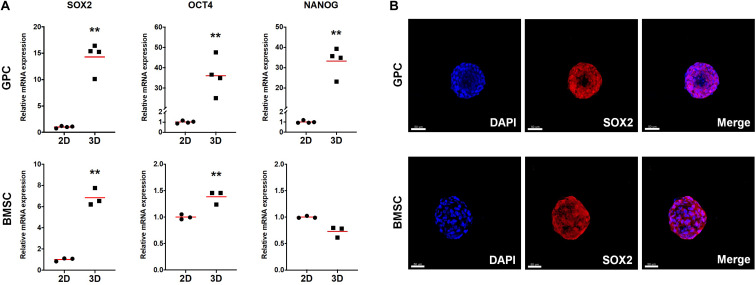
Expression of stemness markers in xeno-free 3D spheroids. **(A)** Relative expression (fold changes) of stemness-related genes after 7 days in 2D and 3D GPCs and BMSCs. Data represent means (*n* = ≥ 3 experimental replicates); statistical analyses are based on delta-Ct values; *p < 0.05; **p < 0.001. **(B)** IF staining of SOX2 in 3D spheroids after 10 days of suspension culture; cell nuclei are stained in DAPI: scale bars 50 μm.

The expression of stemness- and osteogenesis-related genes was assessed in 3D and 2D GPCs and BMSCs after 7 days of suspension culture. SOX2 and octamer-binding transcription factor 4 (OCT4) were significantly upregulated in GPC/BMSC spheroids vs. monolayers (*p <* 0.05); nanog homeobox factor (NANOG) was upregulated only in GPC spheroids (Figure 4A). A relatively higher degree of gene upregulation was observed in spheroids of GPCs as compared to BMSCs. SOX2 and OCT4 were also upregulated in independent donor GPC and BMSC spheroids (Supplementary Figure 2A). Expression of SOX2 in 3D GPCs and BMSCs was confirmed via IF staining (Figure 4B).

**FIGURE 5:**
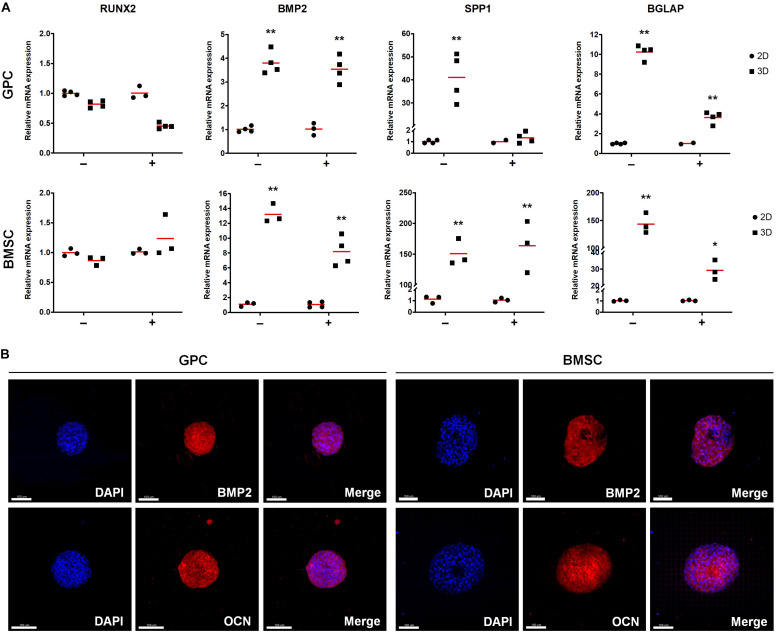
Expression of osteogenesis markers in xeno-free 3D spheroids. **(A)** Relative expression (fold changes) of osteogenesis-related genes after 7 days in 2D and 3D GPCs and BMSCs under non-induced (–) and osteogenically induced conditions (+). Data represent means (*n* = ≥ 3 experimental replicates); statistical analyses are based on delta-Ct values; *p < 0.05; **p < 0.001. **(B)** IF staining of BMP2 and OCN in 3D spheroids after 14 days of suspension culture; cell nuclei are stained in DAPI: scale bars 100 μm.

With regards to osteogenesis, genes associated with both early (BMP2) and late stages [OCN/BGLAP, osteopontin (OPN/SPP1)] of osteogenic differentiation were upregulated in 3D GPCs and BMSCs (*p <* 0.05) (Figure 5A); RUNX2 was upregulated in independent donor, but not pooled, spheroids (Supplementary Figure 2B). In contrast to stemness-related genes, a relatively higher degree of upregulation of osteogenesis-related genes was observed in 3D BMSCs as compared to GPCs. With regards to the effects of osteogenic induction, although BMP2, OPN and OCN were also significantly upregulated in 3D GPCs and BMSCs vs. monolayers after 7 days of osteogenic induction, upregulation of these genes was relatively higher in non-induced spheroids (Figure 5A). Protein expression of BMP2 and OCN after 14 days was confirmed via IF staining (Figure 5B, Supplementary Figure 3); expression of BMP2 was further confirmed via western blotting (Supplementary Figure 4).

**FIGURE 6:**
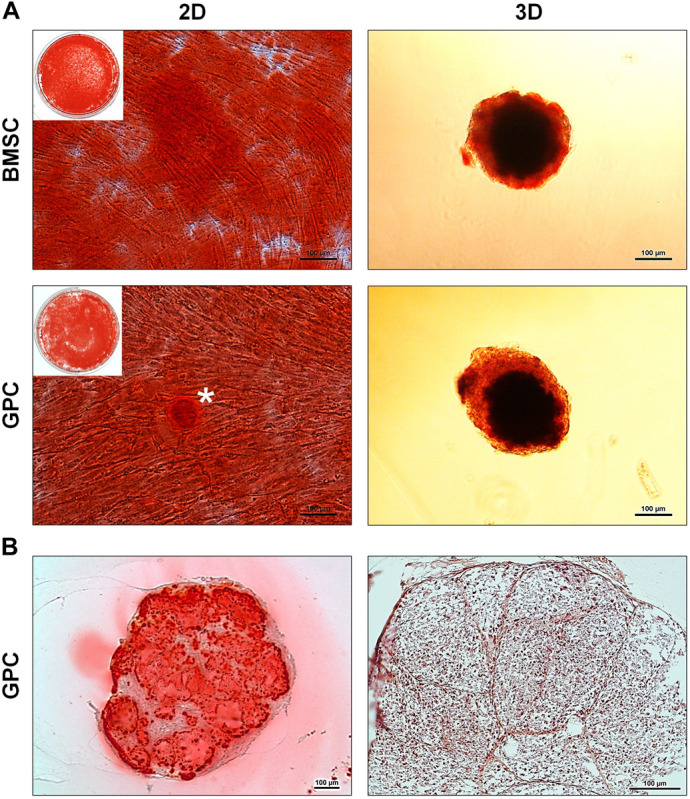
Osteogenic differentiation of xeno-free 3D spheroids. **(A)** Alizarin red staining of 2D and 3D GPCs and BMSCs after 21 days of osteogenic induction; * indicates pronounced mineralization in an area of cellular condensation. **(B)** Histological sections of differentiated 3D GPCs showing internal mineralization (left) and ECM organization following removal of the stain (right); scale bars 100 μm.

After 21 days of osteogenic induction, 3D and 2D GPCs and BMSCs were positively stained for mineral deposition with Alizarin red (Figure 6A). In 2D cultures, the staining appeared to be marginally more intense in BMSCs, while in 3D cultures, the staining appeared comparable between GPC and BMSC spheroids. Mineral staining within the core of the spheroids was confirmed via histology, revealing a mature and organized ECM (Figure 6B).

#### Cytokine Profile of 3D Spheroids

**FIGURE 7:**
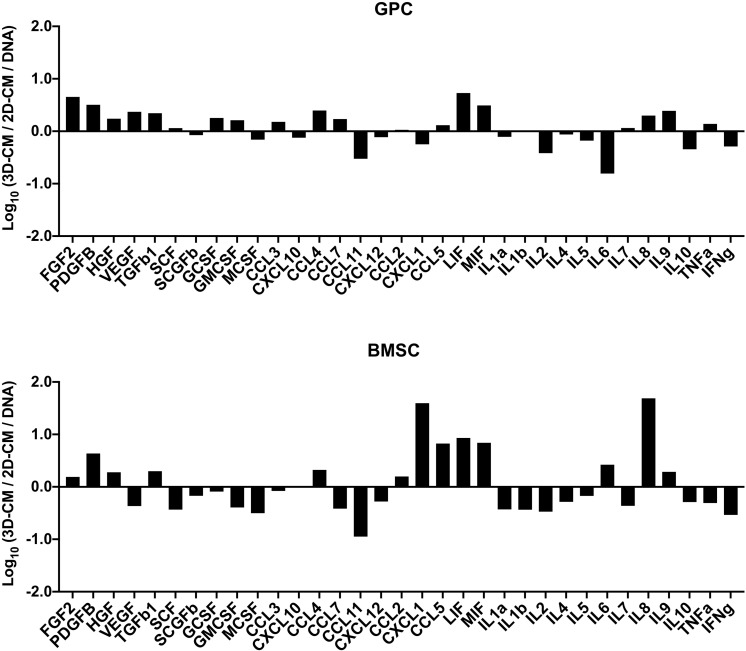
Cytokine profile of xeno-free 3D spheroids. Cytokine concentrations (pg/mL) were measured in the 48 h CM of 2D and 3D GPCs and BMSCs and normalized to their DNA contents (ng/mL). Data are presented as the logarithm (log_10_) of the ratio between the normalized means of 3D-CM and 2D-CM.

The concentrations of various growth factors, chemokines and inflammatory cytokines (Supplementary Table 2) were measured in the 48 h CM of spheroid and monolayer GPCs and BMSCs. Several growth factors (FGF2, PDGF-BB, TGF-β1, HGF, SCF, GCSF) were elevated in spheroid cultures; VEGF was elevated in GPC, but not BMSC spheroids (Figure 6). Notably, both spheroid and monolayer GPCs and BMSCs produced high concentrations of SCGF-β. A number of chemokines (CCL2, CCL3, CCL4, CCL5/RANTES, LIF, MIF) were also elevated in the CM of spheroid GPCs, while others (CCL11, CXCL10, CXCL12) were higher in monolayers; CXCL1 was markedly elevated in the CM of BMSC spheroids. Interestingly, several pro-inflammatory cytokines (IL-1α, 1IL-1β, IL-2, TNF-α, IFN-γ) were downregulated in the CM of GPC and BMSC spheroids, while IL-8 was markedly elevated, especially in BMSCs. The anti-inflammatory IL-10 was upregulated in monolayers in both GPCs and BMSCs (Figure 7).

## Discussion

Gingiva represents a minimally invasive source of multipotent progenitor cells (GPCs) with promising potential for BTE (Wang et al., 2011). To facilitate the clinical translation of GPCs, it is important to characterize their properties in xeno-free cultures compliant with current Good Manufacturing Practices (cGMP). Although previous studies have reported xeno-free culture of cells from other oral tissues using HPL (Naveau et al., 2011; Chen et al., 2012; Wu et al., 2017), to our knowledge, no studies have yet reported on HPL-cultured GPCs. In the present study, GPCs from matched donors were cultured in HPL- or FBS-supplemented media, thus allowing true and standardized comparisons between xeno-free and xenogeneic cultured cells. Overall, the GPCs herein demonstrated superior proliferation and osteogenic differentiation in HPL-supplemented media.

Monolayer GPCs demonstrated a ‘classical’ MSC-immunophenotype (Dominici et al., 2006) with no remarkable differences between HPL- and FBS-cultured cells. However, the specificity of the ‘classical’ surface markers to identify true MSC fractions in heterogeneous cell populations, especially those not derived from bone marrow, has been questioned (Halfon et al., 2011; Lv et al., 2014). CD271 or low-affinity nerve growth factor receptor (LNGFR) is reportedly a more specific marker for isolating a primitive subset of BMSCs with high clonogenicity and multi-lineage, specifically osteogenic, differentiation potential (Cuthbert et al., 2015). Osteogenic enrichment has also been reported in CD271^+^ subsets (< 5%) of dental pulp (DPCs) (Alvarez et al., 2015a) and PDL cells (PDLCs) (Alvarez et al., 2015b). Indeed, a small fraction (1–3%) of CD271^+^ cells was identified in HPL- and FBS-cultured GPCs herein. Interestingly, these cells acquired a neuronal-like morphology; cells in HPL appeared more differentiated with limited proliferation capacity and more homogenous expression of neuronal/glial markers vs. FBS-cultured cells. Indeed, CD271 is reported to be a marker of neural stem/progenitor cells (van Strien et al., 2014). Moreover, craniofacial tissues, including gingiva, have a neural crest origin and therefore contain a subpopulation of cells with the capacity for neurogenic differentiation (Xu et al., 2013). Previous studies have reported the neuronal differentiation of unsorted GPCs when stimulated with neurogenic supplements (Subbarayan et al., 2017; Gugliandolo et al., 2019), although which fraction of the total GPC population actually differentiated, and to what extent, is unclear. Based on the findings herein, the CD271+ GPCs may represent a subpopulation with a propensity for neurogenic differentiation, which is further enhanced in HPL culture. In context, a recent study reported enhanced survival and differentiation of neuronal precursor cells in HPL (Nebie et al., 2020). However, further research is needed to confirm the phenotype and neurogenic potential of CD271+ GPCs.

Concerning multi-lineage differentiation, both HPL- and FBS-cultured monolayer GPCs could be differentiated into adipocytes and osteoblasts *in vitro*. The osteogenic differentiation of GPCs was significantly enhanced in HPL vs. FBS cultures at early and terminal stages, as revealed by gene expression and calcium deposition, respectively. Similar findings have been reported in relation to HPL-cultured DPCs (Chen et al., 2012) and PDLCs (Abuarqoub et al., 2015). Interestingly, the expression of osteogenic genes was also upregulated in non-induced HPL-cultured GPCs after 7 days. It may be hypothesized that this upregulation is related to the presence of several cytokines in HPL, which may influence MSCs’ osteogenic differentiation (Shanbhag et al., 2017). HPL-cultured GPCs also demonstrated attachment and mineralization on copolymer scaffolds, in a comparable manner to BMSCs, highlighting their relevance for BTE applications. Regarding their *in vivo* mineralization capacity, previous studies have reported variable results using FBS-cultured GPCs, ranging from well- to poorly-mineralized tissues (Fournier et al., 2010; Tomar et al., 2010; Wang et al., 2011; Ge et al., 2012; Yang et al., 2013; Moshaverinia et al., 2014). Whether HPL culture enhances the *in vivo* mineralization of monolayer GPCs, remains to be determined.

To overcome the limitations of traditional 2D/monolayer cultures, several studies have demonstrated the benefits of 3D spheroid cultures in terms of promoting the self-renewal, differentiation and paracrine/immunomodulatory activity of MSCs (Murphy et al., 2014; Sart et al., 2014; Follin et al., 2016). Various methods for spheroid culture have been reported (Sart et al., 2014), and can broadly be categorized as *mesenspheres* or *aggregates*. In the *mesenspheres* approach, sphere formation occurs via self-renewal of primary non-expanded (Isern et al., 2013) or early-passage expanded MSCs (Kuroda et al., 2010) seeded in low-density non-adherent cultures. These sphere-forming cells represent ‘true’ stem cells with a capacity for self-renewal and differentiation both *in vitro* and *in vivo* (Basu-Roy et al., 2010; Isern et al., 2013). A small fraction of passage one GPCs herein demonstrated the capacity to form mesenspheres in HPL media. However, the frequency of sphere-forming GPCs was low and of a heterogeneous nature compared to that of BMSCs under similar conditions. One explanation for the low frequency of mesenspheres could be the media composition; mesenspheres have previously only been generated in complex media formulations (Isern et al., 2013) in comparison to the standard HPL media used herein. Nevertheless, obtaining clinically relevant MSC numbers may be challenging with this approach, especially from tissues other than bone marrow.

In contrast to mesenspheres, the more common *aggregates* approach utilizes monolayer expanded cells to form 3D spheroids, either via self-assembly (Baraniak and McDevitt, 2012; Bartosh and Ylostalo, 2014) or forced aggregation (Iwasaki et al., 2019). In the present study, aggregate spheroids were generated via ‘guided’ self-assembly in novel microwell-patterned tissue culture plates – no studies have yet reported this particular micro-well design to generate MSC spheroids. Spheroids with controlled size and morphology were formed after 24 h and showed favorable cell viability with few dead cells after 7 days in HPL-supplemented media. Self-assembly of cells has been linked to events during organogenesis, e.g., MCC during skeletal development (Hall and Miyake, 2000). MCC is known to be a critical event during endochondral ossification and these condensations represent “the earliest sign of the initiation of a skeletal element or elements” (Hall and Miyake, 2000). Indeed, aggregate cultures are routinely used to induce chondrogenic differentiation of MSCs *in vitro*, and often show signs of ‘hypertrophy’ suggestive of endochondral ossification. Even in osteogenically differentiated monolayer MSCs, mineral deposition is observed most prominently in regions of high cellular ‘confluence’ or condensation (Figure 6), after prolonged (2–4 weeks) *in vitro* culture (Kaul et al., 2015). Aggregates of MSCs/osteoprogenitors are reported to mimic such condensations *in vitro*, thereby recapitulating embryonic events during endochondral ossification (Kale et al., 2000; Kim and Adachi, 2019). Moreover, the cytoskeletal changes induced by self-assembly of MSCs into 3D structures, as reviewed elsewhere (Sart et al., 2014), induce “epigenetic” changes which enhance their self-renewal and differentiation potential (Guo et al., 2014).

In *pluripotent* embryonic stem cells (ESCs), self-renewal and maintenance of pluripotency are regulated by three main transcription factors – SOX2, OCT4 and NANOG (He et al., 2009). In *multipotent* cells, such as MSCs, these factors are associated with self-renewal (or ‘stemness’) and maintenance of an undifferentiated cellular state, even in 2D/monolayer cultures (Kolf et al., 2007). In more differentiated 2D cells, e.g., fibroblasts, *ectopic* (over)expression of pluripotency factors triggers cellular reprogramming back to a pluripotent state, as in induced pluripotent stem cells (iPSCs) (He et al., 2009). However, simply changing the microenvironment from 2D to 3D/spheroid culture is known to cause an *intrinsic* upregulation of pluripotency factors in MSCs/osteoprogenitors, suggesting enhanced self-renewal and differentiation potential (Basu-Roy et al., 2010; Guo et al., 2014). Consistently, a significant upregulation of pluripotency factors was observed in 3D vs. 2D GPCs and BMSCs herein. Interestingly, similar observations were recently reported in PDLCs (Moritani et al., 2018) and dermal fibroblasts (Lo et al., 2019). In the latter study, transcriptome analyses revealed differential regulation of 3304 genes in 3D vs. 2D cultures, and the authors concluded that even in naturally heterogeneous populations, such as fibroblasts, the mere shift from a 2D to 3D microenvironment induces gene expression patterns suggestive of “dedifferentiation” or “reprogramming” towards pluripotency (Lo et al., 2019). Both PDL and gingiva are connective tissues with large fibroblast populations. Indeed, fibroblasts from various tissues, including gingiva, are reportedly indistinguishable from MSCs *in vitro*, based on the current “minimal criteria” (Mostafa et al., 2011; Denu et al., 2016). This identical pattern of pluripotency gene-upregulation further supports the evidence for a certain plasticity between ‘MSCs’ and more differentiated cells (Ichim et al., 2018). However, whether upregulation of pluripotency factors in 3D spheroids of GPCs directly translates to enhanced *in vivo* survival, requires further investigation.

In addition to pluripotency markers, an upregulation of early (RUNX2, BMP2) and late osteogenesis-related genes (OPN, OCN) was observed in GPC/BMSC spheroids, even in the absence of osteogenic supplements. As already discussed, a similar upregulation of osteogenic genes was observed in non-induced *HPL*-cultured 2D GPCs. However, *post hoc* analyses of *FBS-*cultured GPC spheroids revealed a similar pattern of osteogenic gene upregulation (Supplementary Figure 5), suggesting that this was primarily an effect of 3D culture. In context, a recent study reported upregulation of osteogenesis-related genes in FBS-cultured spheroids of murine pre-osteoblastic (MC3T3-E1) cells, where a stronger effect of “cell condensation” than osteogenic induction was highlighted, and attributed to recapitulation of ‘MCC-like’ events (Kim and Adachi, 2019). BMPs, including BMP2, are known to mediate MCC during skeletal development *in vivo* (Hall and Miyake, 2000), and are also well-established regulators of MSC osteogenic differentiation *in vitro*, via both extrinsic and autocrine signaling (Phimphilai et al., 2006). BMP2 is also reported to be among the most strongly upregulated genes in 3D spheroids of MSCs (Potapova et al., 2007; Cesarz et al., 2016) and other cells, e.g., fibroblasts (Lo et al., 2019). A previous study reported the ‘early’ *intrinsic* upregulation of BMP2 in FBS-cultured BMSC spheroids, independent of osteogenic induction, which translated to superior *in vitro* ECM production and mineralization vs. 2D BMSCs (Kabiri et al., 2012). The spontaneous upregulation of other bone-related markers (OPN, OCN), along with BMP2, as observed in the GPC/BMSC spheroids herein, further compliments these reports. OPN and OCN are important bone ECM proteins which subsequently undergo mineralization, and their expression is typically associated with later stages of osteogenic differentiation (Liu and Lee, 2013). However, positive staining (Alizarin red) for mineral deposits was only observed in osteogenically induced GPC/BMSC spheroids herein. Indeed, previous studies have reported superior *in vivo* bone regeneration by osteogenically induced spheroids of human BMSCs (Suenaga et al., 2015), DPCs (Lee et al., 2017) and PDLCs (Moritani et al., 2018), vs. monolayers. Thus, it may be hypothesized that MCC-like assemblies induced by spheroid culture *intrinsically* ‘prime’ MSCs towards osteoblastic commitment, although *extrinsic* signals/supplements may be necessary for terminal differentiation and/or matrix mineralization (Kale et al., 2000; Facer et al., 2005).

It is of relevance to discuss the simultaneous upregulation of pluripotency and osteogenesis-related genes in *in vitro* 3D spheroids, in the context of other literature. A similar observation was reported in a previous study comparing the transcriptome of 2D and 3D BMSCs – genes related to pluripotency (SOX2, OCT4, NANOG) and osteogenesis (BMP2, RUNX2, OPN) were upregulated in 3D BMSCs after 3 days of *in vitro* culture (Potapova et al., 2007). The pluripotency factors SOX2, OCT4 and NANOG are known to meditate somatic cell-reprogramming, and intrinsic BMP-signaling is also involved in the early stages this process (Samavarchi-Tehrani et al., 2010). With regard to 2D MSCs, SOX2 and BMP2 were found to be upregulated in subsets of BMSCs with high self-renewal and differentiation potential (Mareddy et al., 2010). Moreover, in ‘reprogrammed’ BMSCs (via forced expression of SOX2 or NANOG), osteogenic differentiation is enhanced, reportedly via BMP-signaling (Go et al., 2008; Ogasawara et al., 2013). In 3D MSCs, the switch to spheroid culture (without extrinsic supplements) leads to an epigenetic upregulation of not only the pluripotency factors, but also BMP2. BMPs, including BMP2, are known to mediate MCC *in vivo*, and MSC spheroids are considered to be the *in vitro* counterparts of ‘MCC-like’ condensations. In the MSC osteogenic differentiation cascade, BMP2 is a potent autocrine regulator of RUNX2, which in turn regulates the downstream expression of osteoblast-specific markers, e.g., OPN and OCN (Liu and Lee, 2013). Indeed, RUNX2, OPN and OCN were found to be upregulated in 3D GPCs and BMSCs herein. Thus, based on the literature, it may be hypothesized that BMP-signaling may act as a ‘link’ between these two distinct processes, i.e., self-renewal and (osteogenic) lineage commitment (Supplementary Figure 6). The co-existence of self-renewing *stem* cells and more-committed *progenitor* cells is a characteristic feature of the stem cell-*niche* (Kolf et al., 2007; He et al., 2009), which appears to be recapitulated in 3D spheroids. However, the role of BMP2 as hypothesized above was not experimentally confirmed herein, and demands further investigation.

Another advantage of 3D culture is the reported enhancement of MSCs’ paracrine and immunomodulatory activity (Follin et al., 2016). Emerging concepts in BTE highlight paracrine- and immune-modulation as primary mechanisms for MSC-mediated bone regeneration (Pittenger et al., 2019). Consistent with previous reports (Zhang et al., 2012; Miranda et al., 2019), the secretome of GPC/BMSC spheroids was enriched in terms of upregulation of several growth factors and chemokines/immune-modulatory cytokines, and downregulation of several pro-inflammatory cytokines. This could, at least partly, explain the observed *in vivo* benefits of spheroid MSCs in regeneration and inflammation models (Zhang et al., 2012; Miranda et al., 2019). Moreover, the enrichment of several cytokines implicated in MSC recruitment and osteogenic differentiation, suggests that transplantation of HPL-cultured 3D GPCs, or their CM, may induce a favorable *in vivo* host-response. Indeed, the CM of 2D GPCs expanded in FBS (Qiu et al., 2020) or defined serum-free medium (Diomede et al., 2018) has recently been shown to promote *in vivo* bone regeneration. Interestingly, both 2D and 3D GPCs (and BMSCs) herein, secreted high concentrations of stem cell growth factor (SCGF) – a protein encoded by the CLEC11A gene, which has been shown to promote osteogenic differentiation and *in vivo* fracture healing in murine MSC-models (Yue et al., 2016). Since high concentrations of SCGF were also detected in HPL (data not shown), this could be another benefit of HPL supplementation for BTE applications. Finally, whether the combination of HPL supplementation and 3D culture enhances the *in vivo* bone regeneration capacity of GPCs, should be investigated in future studies.

## Conclusion

Monolayer GPCs expanded in HPL vs. FBS demonstrate enhanced *in vitro* osteogenic differentiation, comparable to that of BMSCs. When cultured as 3D spheroids in HPL, both GPCs and BMSCs express significantly higher levels of pluripotency genes as compared to monolayers, suggesting a higher potential for self-renewal. Simultaneously, the expression of osteogenesis-related genes is also significantly increased in GPC and BMSC spheroids, independent of osteogenic induction; *in vitro* mineralization was comparable between GPCs and BMSCs Finally, the secretome of GPC and BMSC spheroids is enriched, in terms of several growth factors, chemokines and immune-modulatory cytokines, in comparison to that of monolayers. In summary, while xeno-free cultured spheroids of GPCs are comparable to BMSCs *in vitro*, GPCs offer the advantage of less-invasive tissue harvesting and are thus promising candidates for BTE applications.

## Data Availability Statement

All datasets presented in this study are included in the article/Supplementary Material. Additional data can be made available by the authors upon request.

## Author Contributions

SSh designed the study, performed the experiments, analyzed the data, and drafted the manuscript. SSu contributed to the design, experiments, data analysis, and manuscript writing. AIB, AS, and KM contributed to the design, data analysis, and manuscript writing. All authors read and approved the final manuscript.

## Conflict of Interest

The authors declare that the research was conducted in the absence of any commercial or financial relationships that could be construed as a potential conflict of interest.
