# Supplementary material for: Xeno-Free Spheroids of Human Gingiva-Derived Progenitor Cells for Bone Tissue Engineering
Source: Front Bioeng Biotechnol. 2020 Aug 19;8:968. doi: 10.3389/fbioe.2020.00968 (PMC7466771; doi:10.3389/fbioe.2020.00968)
Supplement: Supplementary file 1 [file Data_Sheet_1.pdf]

Supplementary material

1. Supplementary methods

1.1. Production of human platelet lysate (HPL)

The HPL herein (Bergenlys®, Bergen, Norway) was prepared from outdated pooled whole blood-derived platelet concentrates (PCs) at the Department of Immunology and Transfusion Medicine, Haukeland University Hospital, Bergen, Norway. Briefly, unused 7 d-old PCs were used for HPL production via multiple freezing (–80°C) and thawing cycles (+37°C). Pooled PCs were then centrifuged at 3000 x g (4°C, 15 min) to remove platelet fragments and aliquoted as the final HPL product. HPL aliquots were stored at –80°C and thawed overnight at 4°C for subsequent use in experiments. In HPL-supplemented media, 1 IU/mL of heparin was added to prevent gelation and the medium was sterile filtered (0.2 µm) before use.

2. Supplementary tables and figures

2.1. Supplementary table 1: Real-time PCR assays

**Gene**	**TaqMan® Assay ID**	**Amplicon length**
**Housekeeping gene**		
GAPDH	Hs 02758991_g1	93
**Adipogenesis-related**		
PPARG	Hs01115513_m1	90
LPL	Hs00173425_m1	103
**Osteogenesis-related**		
RUNX2	Hs01047973_m1	86
BMP2	Hs00154192_m1	60
ALPL	Hs01029144_m1	79
COL1A2	Hs00164099_m1	68
OPN (SPP1)	Hs00959010_m1	84
OCN (BGLAP)	Hs01587814_g1	138
**Stemness-related**		
SOX2	Hs01053049_s1	91
OCT4 (POU5F1)	Hs00999632_g1	77
NANOG	Hs02387400_g1	109

GAPDH glyceraldehyde 3-phosphate dehydrogenase, PPARG peroxisome proliferator activated receptor gamma, LPL lipoprotein lipase, RUNX2 runt-related transcription factor 2, BMP2 bone morphogenetic protein 2, ALPL alkaline phosphatase, COL1A2 Collagen type 1, OPN/SPP1 Osteopontin, OCN/BGLAP Osteocalcin, SOX2 sex determining region Y-box 2, OCT4/POU5F1 octamer-binding transcription factor 4, NANOG homeobox transcription factor nanog

Supplementary material

2.2. Supplementary table 2: Multiplex human cytokine screening panel

Abbreviation	Cytokine
b-FGF/FGF2	Basic fibroblast growth factor
Eotaxin/CCL11	C-C chemokine 11
G-CSF	Granulocyte colony stimulating factor
GM-CSF	Granulocyte-macrophage colony-stimulating factor
IFN-γ	Interferon-γ
IL-1β	Interleukin-1β
IL-1ra	Interleukin 1 receptor antagonist
IL-1α	Interleukin-1α
IL-2Rα	Interleukin-2 receptor α
IL-3	Interleukin-3
IL-12 (p40)	Interleukin-12 subunit beta
IL-16	Interleukin-16
IL-2	Interleukin-2
IL-4	Interleukin-4
IL-5	Interleukin-5
IL-6	Interleukin-6
IL-7	Interleukin-7
IL-8	Interleukin-8
IL-9	Interleukin-9
GRO-α/CXCL1	Growth-regulated alpha protein/CXC ligand 1
HGF	Hepatocyte growth factor
IFN-α2	Interferon-α2
LIF	Leukemia inhibitory factor
MCP-3/CCL7	Monocyte chemotactic protein-3
IL-10	Interleukin-10
IL-12 (p70)	Interleukin-12
IL-13	Interleukin-13
IL-15	Interleukin-15
IL-17A	Interleukin-17
IL-18	Interleukin-18
IP-10/CXCL10	Interferon gamma-induced protein 10/CXC chemokine 10
MCP-1/CCL2	Monocyte Chemoattractant Protein-1
MIG/CXCL9	Monokine induced by gamma interferon/CXC ligand 9
β-NGF	Nerve growth factor
SCF/KITLG	Stem cell factor/KIT-ligand
SCGF-β	Stem cell growth factor
SDF-1α/CXCL12	Stromal cell-derived factor 1
MIP-1α/CCL3	Macrophage inflammatory protein
MIP-1β/CCL4	Macrophage inflammatory protein
PDGF-BB	Platelet-derived growth factor-BB
RANTES/CCL5	Regulated on activation, normal T cell expressed and secreted
Eotaxin/CCL11	Eotaxin
TNF-α	Tumor necrosis factor-α
TNF-β	Tumor necrosis factor-β
VEGF	Vascular endothelial growth factor
CTACK/CCL27	Cutaneous T-Cell Attracting Chemokine
MIF	Macrophage migration inhibitory factor
TRAIL	TNF-related apoptosis-inducing ligand
M-CSF	Macrophage colony-stimulating factor

Supplementary material

2.3. Supplementary figure 1: Osteogenic differentiation of monolayer GPCs and BMSCs on copolymer scaffolds

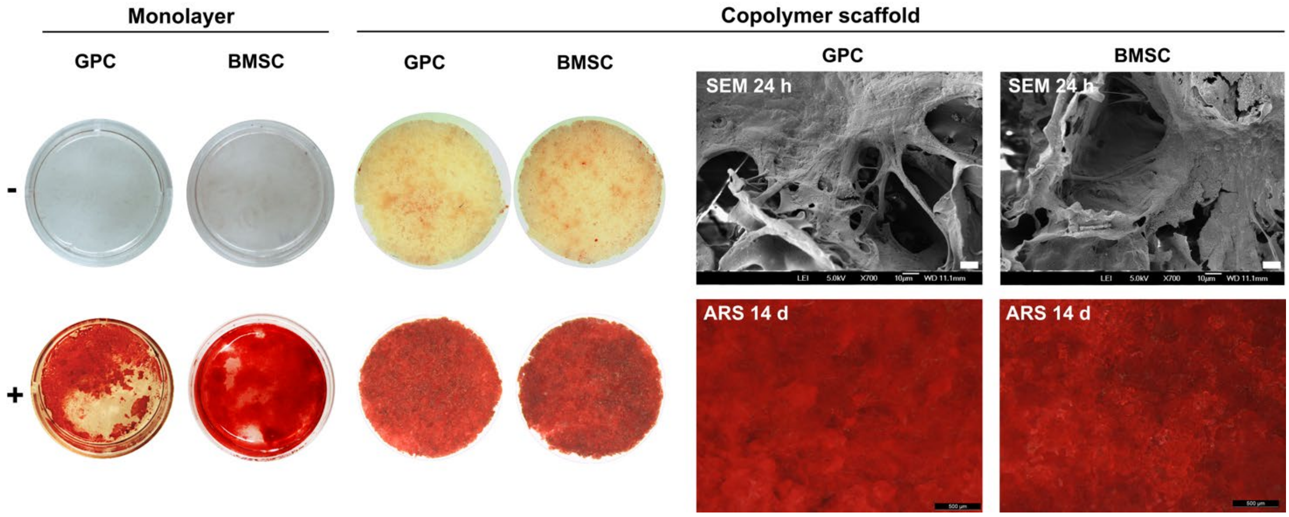

Alizarin red S staining (ARS) reveals osteogenic differentiation of GPCs and BMSCs in monolayer (21 d) and on poly(LLA-co-CL) scaffolds (14 d) under osteogenically induced (+) and non-induced (–) conditions; SEM reveals cell attachment and spreading on the scaffold surface after 24 h; scale bars 10 µm (*top*); magnified view of Alizarin red-stained scaffolds (*bottom*, scale bars 500 µm)

Supplementary material

2.4. Supplementary figure 2: Gene expression in 3D spheroids of independent donors.

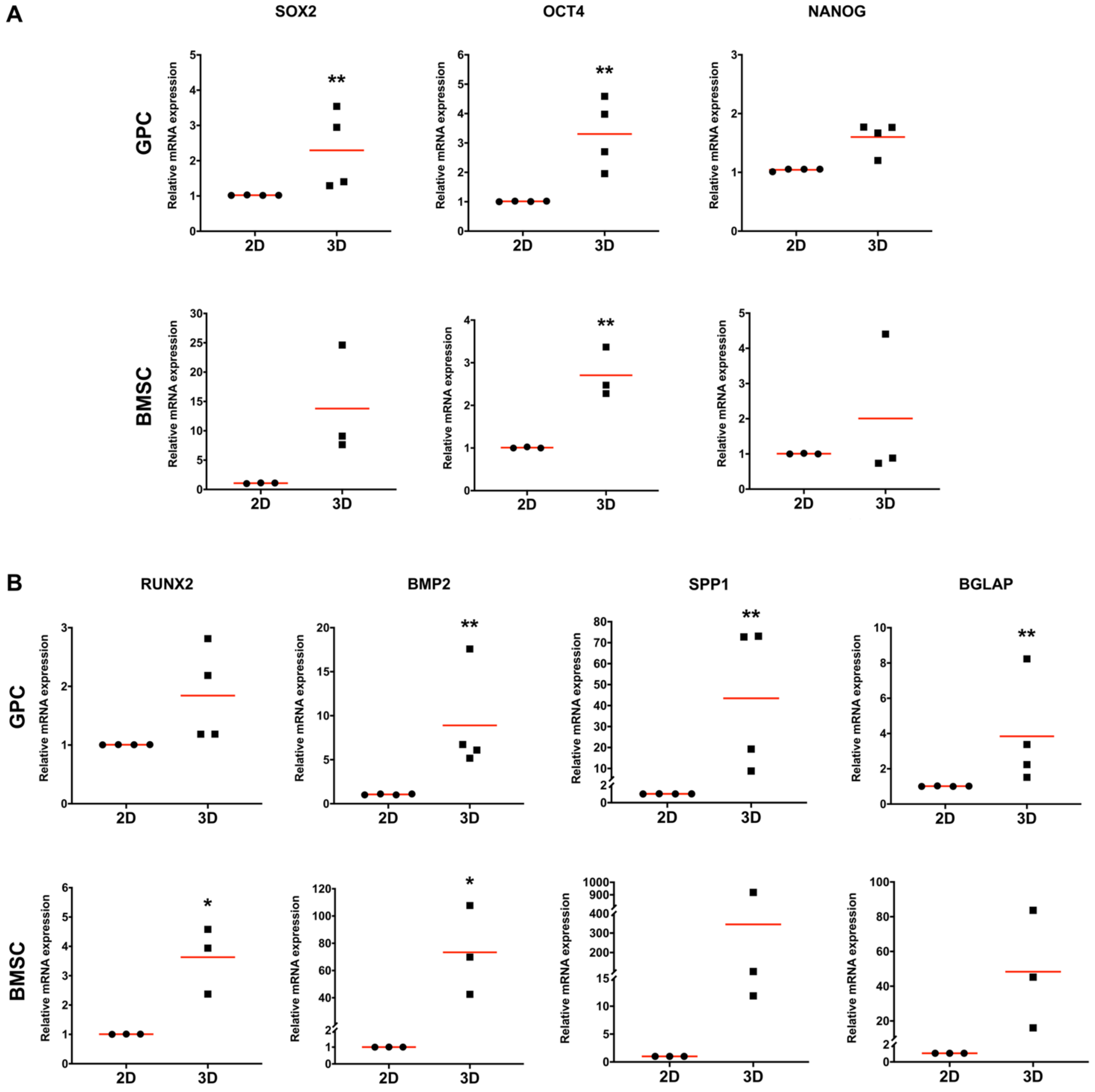

Relative expression (fold changes) of stemness- (A) and osteogenesis-related genes (B) after 7 d in 2D vs. 3D GPCs and BMSCs; data represent means; each symbol represents a single donor (n=>3); statistical analyses are based on delta-Ct values; *$p < 0.05$; **$p < 0.001$.

2.5. Supplementary figure 3: Immunofluorescence staining of 2D GPCs and BMSCs

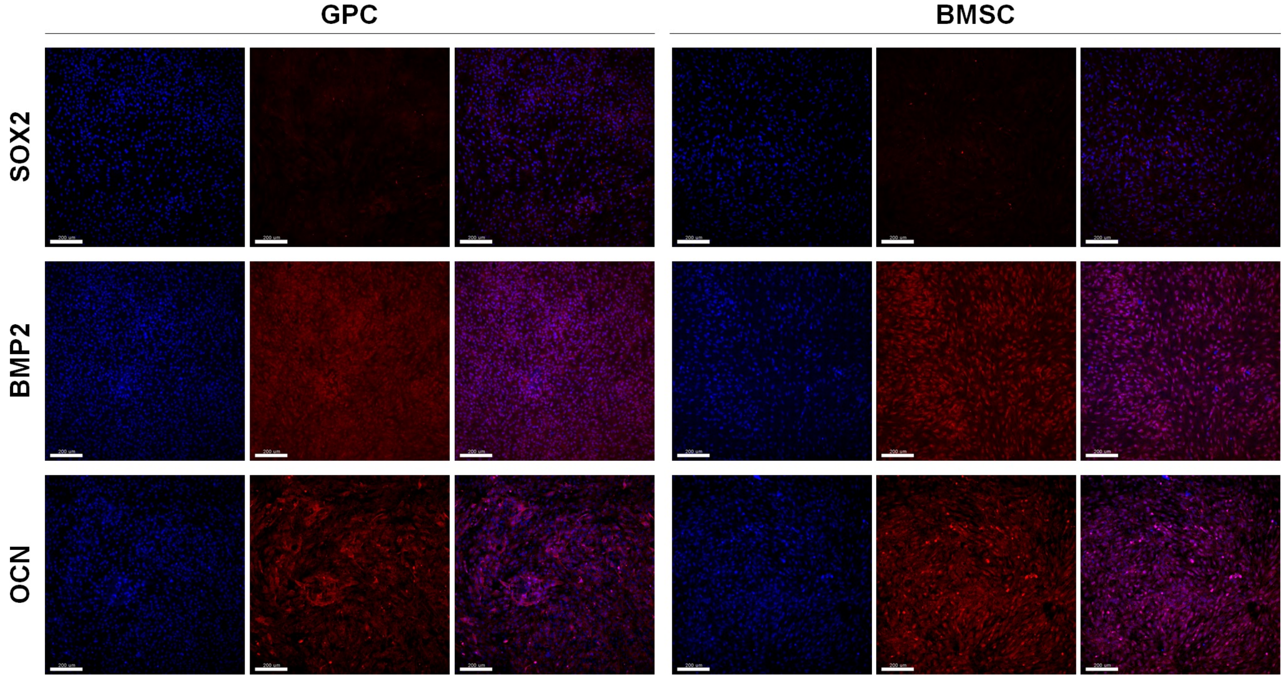

IF staining of 2D monolayer GPCs and BMSCs for stemness (SOX2) and osteogenesis-related markers (BMP2, OCN) after 14 d culture in standard and osteogenic induction media, respectively; scale bars 100 μm. Positive and comparable staining for BMP2 and OCN was observed in GPCs and BMSCs.

Supplementary material

2.6. Supplementary figure 4: Protein estimation of BMP2 via western blotting in 2D and 3D GPCs and BMSCs

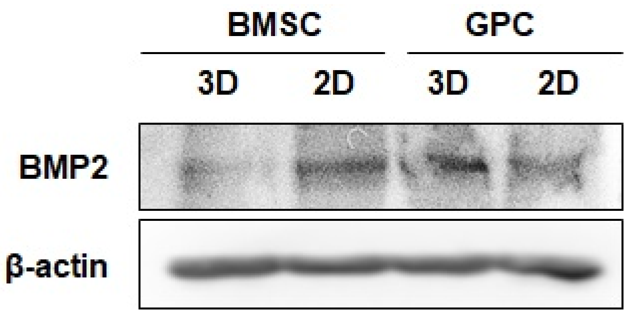

Protein estimation of BMP2 was performed via western blotting via SDS gel electrophoresis and blotting onto polyvinylidene difluoride (PVDF) membranes. The membranes were incubated with the following primary antibodies overnight at 4°C: anti-BMP2 (R&D systems) and anti-β-actin as a reference protein (Invitrogen). After extensive washing, the membranes were further incubated with horseradish peroxidase-conjugated secondary antibodies for 1 h and then developed using an enhanced chemiluminescence detection system (all from Bio-rad).

2.7. Supplementary table 3: Cytokine concentrations in 2D and 3D GPCs and BMSCs.

Supplementary material

	GPC				BMSC			
	2D		3D		2D		3D	
	Mean	SD	Mean	SD	Mean	SD	Mean	SD
**Growth factors**								
FGF2	0.04012	0.00478	0.18143	0.00197	0.02768	0.00530	0.04271	0.00001
PDGFbb	0.04337	0.01677	0.13879	0.00952	0.03152	0.00506	0.13655	0.00067
HGF	24.24730	0.87560	42.10489	1.40289	1.99578	0.25363	3.77988	0.06782
VEGF	0.72088	0.12111	1.69592	0.04196	3.94904	0.43366	1.69724	0.05659
TGFb1	24.50784	25.92031	53.98704	6.14884	17.95800	1.87240	35.67928	6.78256
SCF	0.04295	0.00382	0.04931	0.00303	0.05008	0.00406	0.01840	0.00024
SCGFb	113.14256	3.54892	95.36207	2.94256	110.21860	8.45354	74.49511	1.97597
GCSF	0.25382	0.03767	0.45470	0.03915	0.16630	0.00391	0.13473	0.00454
GMCSF	0.00281	0.00422	0.00456	0.00021	0.00202	0.00021	0.00082	0.00012
MCSF	0.03043	0.00432	0.02100	0.00062	0.04031	0.00780	0.01267	0.00030
**Chemokines**								
CCL11	0.16741	0.00556	0.04998	0.00012	0.01049	0.00137	0.00118	0.00003
CXCL10	0.03273	0.00471	0.02468	0.00072	0.00911	0.00330	0.00907	0.00001
CCL2	2.13776	0.13538	2.27424	0.07038	0.93586	0.12059	1.47786	0.02184
CCL3	0.00086	0.00029	0.00129	0.00001	0.00088	0.00023	0.00073	0.00005
CCL4	0.04168	0.01010	0.10332	0.00365	0.03960	0.01123	0.08331	0.00226
CCL5	5.85575	0.20835	7.63716	0.15172	1.44246	0.22004	9.65767	0.20100
CXCL1	3.65130	0.01579	2.06174	0.13972	0.16873	0.03515	6.66148	0.41839
CCL7	0.08885	0.00569	0.15191	0.00706	0.53342	0.06061	0.20476	0.00486
LIF	0.12380	0.02919	0.66383	0.01056	0.07635	0.01774	0.65338	0.00975
MIF	1.30814	0.07396	4.06624	0.26745	0.72819	0.29118	5.04052	0.25619
CXCL12	0.23595	0.02569	0.18201	0.00560	0.21189	0.03144	0.11132	0.00479
**Inflammatory cytokines**								
IL1a	0.02693	0.00994	0.02109	0.00164	0.02588	0.00858	0.00960	0.00001
IL1b	0.00251	0.00102	0.00247	0.00001	0.00222	0.00067	0.00081	0.00011
IL2	0.00471	0.00280	0.00180	0.00001	0.00324	0.00223	0.00109	0.00032
IL4	0.00353	0.00214	0.00307	0.00019	0.00376	0.00068	0.00194	0.00004
IL5	0.05189	0.05215	0.03438	0.00001	0.03339	0.00435	0.02244	0.00091
IL6	4.37381	0.03748	0.68100	0.06986	0.82140	0.10703	2.16931	0.07043
IL7	0.01789	0.00672	0.02066	0.00310	0.01402	0.00315	0.00608	0.00083
IL8	0.31796	0.06139	0.63340	0.07276	0.07324	0.01182	3.57365	0.03481
IL9	0.06582	0.02222	0.16124	0.00506	0.06258	0.01968	0.12090	0.00044
IL10	0.00854	0.00080	0.00387	0.00001	0.00502	0.00252	0.00256	0.00033
TNFa	0.01963	0.00316	0.02714	0.00001	0.02310	0.00592	0.01133	0.00123
IFNg	0.07505	0.00856	0.03856	0.00394	0.06054	0.00678	0.01762	0.00123

To account for differences in cell numbers between 2D and 3D cultures at the end of the culture period, cytokine concentrations (pg/mL) were normalized to the corresponding total DNA (ng/mL); data represent pg protein/ng DNA.

Supplementary material

2.8. Supplementary figure 5: Gene expression in 3D spheroids of FBS-cultured GPCs.

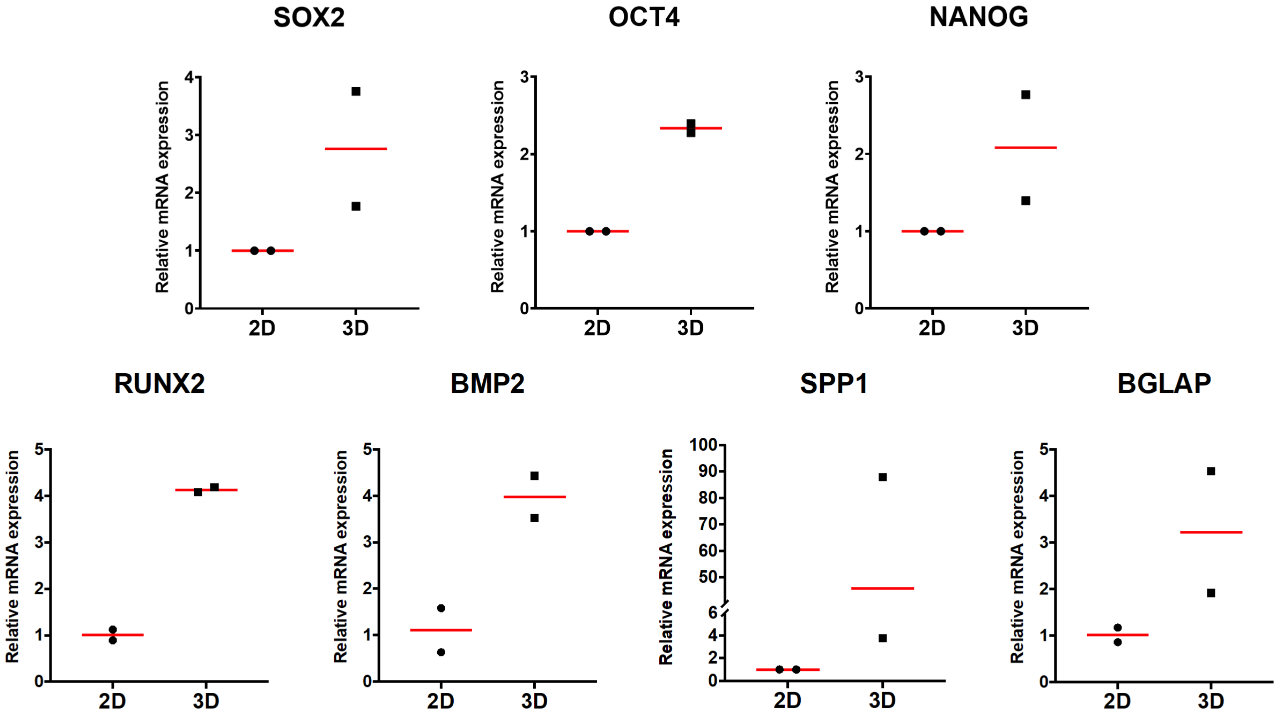

Relative expression (fold changes) of stemness- and osteogenesis-related genes after 7 d in 2D vs. 3D GPCs; data represent means; each symbol represents a single donor (n=2).

Supplementary material

2.9. **Supplementary figure 6: Schematic representation of the hypothesized mechanism for self-renewal and lineage commitment in 3D spheroids.**

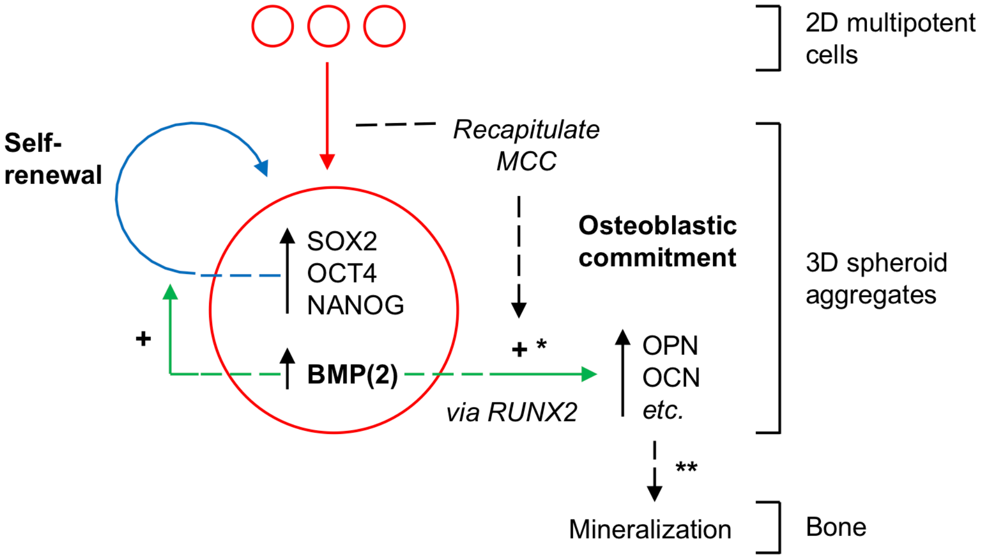

MCC, mesenchymal cell condensations; * independent of osteogenic induction; ** dependent on osteogenic induction.
